# Restriction site-associated DNA sequencing (RAD-seq) analysis in Pacific oyster *Crassostrea gigas* based on observation of individual sex changes

**DOI:** 10.1038/s41598-020-67007-4

**Published:** 2020-06-18

**Authors:** Chenyang Yue, Qi Li, Hong Yu, Shikai Liu, Lingfeng Kong

**Affiliations:** 1 Key Laboratory of Mariculture, Ministry of Education, Ocean University of China, Qingdao, 266003 China; 2 Laboratory for Marine Fisheries Science and Food Production Processes, Qingdao National Laboratory for Marine Science and Technology, Qingdao, 266237 China

**Keywords:** Agricultural genetics, Genetic markers

## Abstract

The diverse modes of sexual reproduction in Bivalvia make it an excellent clade to understand the evolution of sex and sex determination. The cosmopolitan Pacific oyster *Crassostrea gigas* is an ideal model for bivalve sex determination studies because of its complicated sexuality, including dioecy, sex change and rare hermaphroditism. A major barrier to *C. gigas* sex determination study has been the lack of information on the type of sex determination. To identify its sex-determining system, sex observation by following the same individual in two consecutive years was conducted on 760 oysters from distinct populations. Stable sexuality and sex reversal in both directions were observed, which provides a case against the protandry of *C. gigas*. Restriction site-associated DNA sequencing (RAD-seq) based on 26 samples with unchanged and converted sexualities was carried out for identifying sex-linked marker. One SNP Cgsl-40 was proved to be sex-related, but sex-biased heterozygosity varied between populations for RAD-seq and validation, showing no evidence for sex chromosomes or single-locus models for *C. gigas* primary sex determination. Information obtained in our study provides novel insight into sex determination mechanism in *C. gigas*.

## Introduction

Sex determination establishes sexual fate of organisms and activates testis or ovarian pathway in a dimorphic manner^1^, which is absolutely fundamental to the sexual reproduction. In general view, gender of organisms is thought to be influenced by genetic, environmental factors or both of them^2,3^, and the various sex determination mechanisms across taxa has long been interest to many biologists. Chromosomal sex determination (CSD) is the most extensively studied mode of genetic sex determination (GSD), such as typical XY/XX system in mammals and ZZ/ZW system in aves, in which sex is determined by a primary switch gene located on one or both members of a well-differentiated chromosomal pair^4,5^. In the polygenic sex determination (PGSD) system, by contrast, the sex determination genes are distributed across the genome and the combination of their alleles determines the sex of individual, in which sex behaves as a threshold quantitative trait^5–7^. Environmentally-governed sex determination (ESD) is another type of sex determination in the animal kingdom, and the environmental factors, such as temperature, affect sex determination during a critical time window in such systems^8–10^. Some species, such as zebrafish *Danio rerio*^5,11^ and European sea bass *Dicentrarchus labrax*^12^, have a mixture of PGSD and ESD, but this form of sex determination has not been studied extensively as CSD at the experimental level.

Although studies in model organisms have revealed key genes and complex pathways involved in sex determination and differentiation, understanding the evolution of sex requires more information about molecular mechanisms of sex determination in diverse taxa, especially invertebrates with varying sexuality. The Bivalvia contributes the major branch of the second largest animal phylum Mollusca and diverse modes of sexual reproduction have been found, ranging from functional (simultaneous) hermaphroditism, alternative sexuality, to strict dioecy^13–15^. This makes it an excellent clade to investigate one of most fundamental transitions in sex determination from hermaphroditism to gonochorism. But there is limited knowledge on this subject. One bivalve species, the dwarf surfclam *Mulinia lateralis*, has already been suggested to have a XY/XX system^16^. Whereas, the determinants of sex in many bivalve species studied to date appear to be both genetic and environmental^10,15,17,18^. For example, female-biased sex ratio at high temperature was observed in natural populations of *Crassostrea virginica*^19^. Controlled crosses of *C. virginica* suggested that sex in this species was also under genetic control and a three-loci model for sex determination was proposed^20,21^.

The Pacific oyster, *Crassostrea gigas*, is an ideal model with alternative sexuality to understand the sex determination in bivalves. *C. gigas* is dioecious without secondary sex characteristics, which means it is difficult to identify the sex of oyster from its appearance. And the most interesting is that *C. gigas* can change sex in both directions between different reproductive seasons^22,23^. Furthermore, low frequencies simultaneous hermaphrodites can also be observed^13,14^. For years, researchers mainly focused on several kinds of studies to understand genetic mechanisms of *C. gigas* sex determination, such as sex ratio studies^14,24,25^, identification/isolation of sex-related DNA markers^26^, and surveys and comparisons of genomes and/or transcriptomes^27,28^. Studies employing controlled crosses provided evidence for paternal control of sex in *C. gigas*^14^. Some researchers proposed that sex of *C. gigas* was determined by two-genotypes at a single locus with FM individuals always being males and FF individuals being either males or females^14^. An alternative three-genotype model was generated with fixed MM males and two kinds of females, fixed FF and protandric FM; the model involves an “f” parameter, which is the probability that FM individuals mature as females, explaining heterogeneity in sex ratios^25^. In addition, environmental factors, such as exogenous steroids, temperature and food availability were also thought to affect sex ratios in *C. gigas*^10,24^. Recently, the genetic factors of sex determination were studied in more details. Up to now, some downstream actors of the molecular cascade of the Pacific oyster sex determination, such as *Cg-β-catenin*, *CgFoxl2*, *Cg-SoxE* and *CgDsx*, have been characterized^10,27–29^, and several QTLs for sex were detected in linkage mapping^25,26^, while no major sex-determining gene or genetic marker supporting above two genetic models has been identified.

The most direct search for sex determination mechanism is to look for genomic differences between the male and female^11^, and the ongoing rise of next-generation sequencing is now opening a new chapter in sex determination research^30^. Restriction site-associated DNA sequencing (RAD-seq) was applied successfully in identifying a male-specific marker based on several field-collected *Anolis carolinensis* individuals^31^. Afterwards, various strategies using RAD-seq were proven to be effective in sex-specific markers or sex chromosomes identification in species without heteromorphic sex chromosomes, such as *Hypophthalmichehys nobilis*, *H. molitrix*, *Rana arvalis* and 12 gecko species^30,32,33^. The utility of RAD-seq for identifying sex-determining systems provided an opportunity to increase the knowledge about the Pacific oyster sex determination.

Sex change is a characteristic feature of *C. gigas*, which is responsible for sex ratios varying with age in a fixed group. To understand the sexual system of *C. gigas*, it is essential to employ an analysis of individuals that sexes are observed at multiple reproduction seasons^34^. In this study, the sex of the same Pacific oyster individual at two consecutive sexual maturation stages was observed. Four types of individuals were collected, including those that changed sex in both directions (female-to-male and male-to-female) and those without sex reversal (male-to-male and female-to-female). RAD-seq was employed to identify sex-linked markers among individuals of four phenotypic genders in order to provide more information about the sex determination system of *C. gigas*.

## Materials and methods

### Sex observation and sampling

A total of 760 one-year-old adult Pacific oysters (mean shell height was 60.20 ± 4.23 mm) were collected from hatchery populations (Rushan, Shandong, China, 36.4°N, 121.3°E) during the gonad maturation stage (May and June 2016). After temporary cultivation in capacious tanks for one week, oysters were anaesthetized using filtered seawater with additional 50 g L^−1^ MgCl_2_^35^. For each oyster, a little gonad tissue was extracted to discern its gender under a microscope when shell was opened slightly. After sex recorded, each oyster was put in a separate mesh bag with its gender label then returned to clean seawater. Cultivated in capacious tanks for one week, they were transferred to natural sea until next maturation (May 2017). In the second maturation stage, each individual gender was identified again, after that oysters were sacrificed for tissue sampling, including gonad, adductor, gill and mantle.

### Restriction site-associated DNA sequencing and data processing

In this study, 26 oysters (eight that changed sex from male to female (MF), six that female to male (FM), six males (MM) and six females (FF) without sex reversal) were selected for RAD-seq libraries construction. Briefly, genomic DNA was extracted from adductor muscle of the specified individuals using SDS-phenol-chloroform and digested with *EcoR*I (New England Biolabs). Individually barcoded P1 adapters were ligated onto the *EcoR*I cut site for each sample. Samples were pooled in proportionate amounts for shearing to average size 500 bp. After manually size-selected into 300- to 700-bp fragments by gel electrophoresis, libraries were blunt-end-repaired, and a 3' adenine overhang added to each fragment. We added a P2 adapter containing unique Illumina barcodes for each library. Libraries were amplified via 16 cycles of PCR with Phusion high-fidelity DNA polymerase (New England Biolabs) and column-purified. Samples were sequenced on an Illumina Hiseq 4000 using 150-bp paired-end sequencing. Clean data were obtained through quality trimming, which removed reads with ≥ 10% unidentified nucleotides (N), reads with > 50% bases having Phred quality scores of ≤ 20% and reads aligned to the barcode adapter from raw data generated from Illumina sequencing.

### Variants identification and annotation

*C. gigas* genome assembly (oyster_v9) and gene model annotation files were downloaded from the NCBI (ftp://ftp.ncbi.nlm.nih.gov/genomes/Crassostrea_gigas/). To identify SNPs, the Burrows-Wheeler Aligner (BWA) was used to align the clean reads from each sample against the reference genome with the setting ‘mem 4 -k 32 -M’, in which -k is the minimum seed length and -M is an option used to mark shorter split alignment hits as secondary alignments. Variant calling was performed for all samples using the GATK’s Unified Genotyper. SNPs were filtered using the GATK’s Variant Filtration with proper standards (-Windows 4, -filter “QD < 2.0 | | FS > 60.0 | | MQ < 40.0”, -G_filter “QD < 20”).

### Putative sex-linked markers identification

The strategy to detect sex-linked markers was inspired by an excellent work in identifying homomorphic sex chromosomes from wild-caught adults with limited genomic resources^30^. Based on sex differences in genotype frequencies and sex-limited occurrence, potentially sex-linked markers were filtrated through three types of sample grouping as follows. (1) Samples were divided into two groups according to their gender at the first maturation, FF_FM (six FF and eight FM samples) and MM_MF (six MM and six MF samples). The putative sex-linked markers were identified with homemade Perl scripts under the standard that, for a specific locus, at least 80% samples from one group (allowing for sequencing errors and phenotype classification errors) stay the same genotype and show different compared with samples assigned in another group^30^. Twelve possible genotype differences between two groups were considered, including heterozygosity, homozygosity and indel. (2) Three sample sets, MM, FF and MF_FM (the sum of MF and FM samples), were generated based on samples gender at two sequential sexual maturation. Similar standard was set to identify 66 kinds of loci that dominant genotype of one sample group differed from those of the other two. (3) Sex-linked markers were filtered with similar standard as above two strategies when MM, FF, MF and FM samples were considered separately, and 246 possibilities were included. The third grouping is expected to identify some of the same markers generated by the above two.

### Sex-linked markers validation

Putative sex-linked markers were chosen for further validation according to the read coverage (> 15 ×) and variants structure type. Firstly, PCR was conducted using DNA samples for RAD-seq and the products were Sanger-sequenced. After this, the filtered sex-linked markers that sanger sequencing results kept consistent with RAD-seq, were examined with SNaPshot in additional individuals from another population, including ten MM samples, eleven FF samples, three MF samples and eight FM samples. The population for validation and the population for RAD-seq were cultured in the same oyster farm but came from different parents.

## Results

### Sex observation by following the same individuals

Through years of individual follow-up sex observation on 760 Pacific oysters, sex information of 130 oysters at two consecutive maturation stages was collected (Table 1). Sex change in both directions was observed and the rate of sex reversal was 29.23% (*n* = 38/130). According to the data, 96 individuals exhibited female sex at the first sexual maturation and 20.83% changed from female to male (*n* = 20/96); 34 individuals exhibited male at the first sexual maturation and 52.94% changed from male to female (*n* = 18/34) (Table 1).

**Table 1:** Sex observation by following the same individuals at two consecutive maturation stages.

	Number	Sex change rate for each primary sex	Sex change rate for the entire population
Female to male	20	20.83% (*n* = 20/96)	15.38% (*n* = 20/130)
Female to female	76	79.17% (*n* = 76/96)	58.46% (*n* = 76/130)
Male to male	16	47.06% (*n* = 16/34)	12.31% (*n* = 16/130)
Male to female	18	52.94% (*n* = 18/34)	13.85% (*n* = 18/130)
Total	130	29.23% (*n* = 38/130)	100%

### RAD reads processing

In this study, 26 oysters were selected for RAD-seq and 286,669,572 raw reads were produced by Illumina sequencing. After removing low-quality sequence and ambiguous barcodes, 271,783,556 clean reads were retained with Q30 (%) varying from 92.89% to 95.87%, yielding a mean depth of 8.39 ×(range of 6.90 X to 9.70 ×), and 79.16% of them were aligned to the Pacific oyster genome with an average coverage rate of 17.25% (range of 12.03% to 23.44%) (Table S1). Raw Illumina RAD-seq reads have been deposited in the NCBI Short Read Archive (SRP167922).

### Variants identification

After filtering using the GATK’s Variant Filtration, 8,334,610 SNPs and 945,571 InDel markers totally called by the GATK’s Unified Genotyper were retained and the transition to transversion ration is 1:0.93. Overall, the SNP density was 16.64 per kb, with at least one SNP per 78 kb. Most of these variants fall in the intronic regions of genes (40.11%) and the intergenic regions (37.34%) (Table S2), in addition, the absence of function information of 92.99% variants was detected (Table S3).

### Putative sex-linked markers identification

All of the above 9,280,181 loci were used for sex-linked markers identification. Samples were divided into two groups, FF_FM and MM_MF, according to their gender at the first maturation, and eight loci were filtrated. This grouping identified one locus where most individuals from MM_MF group were heterozygous, while those from FF_FM group were homozygous. There were five loci present in MM_MF group, but absent in the FF_FM group and there were also two loci that showed opposite occurrence with the above five (Table S4).

According to two-genotypes and three-genotypes genetic models for *C. gigas* sex determination^14,25^, individuals were classified into three groups, MM, FF, and MF_FM. There were 94 loci in which majority from one group were heterozygous, while those from the other two groups were homozygous. Fifteen loci were found with opposite genotype variance, which means that for them, individuals from one group were heterozygous and those from the other two groups were homozygous. There were 2,714 loci were identified as sex-limited because of the absence in individuals from one group and presence in those from the other two groups, and 4,095 loci were absent in individuals from two groups but present in those from the remaining one (Table S5).

In the last grouping, four kinds of individuals, MM, FF, MF and FM, were assigned into four uncorrelated sample sets. This grouping identified 85 loci where most individuals from one group were heterozygous, while those from the other three groups were homozygous in them. There were ten loci in which majority of individuals from one set were homozygous, and those from other three were heterozygous. Five loci were also kept because individuals from two groups were homozygous in them and those from other two groups were heterozygous. In addition, there were 138 loci that were absent in individuals from one sample group while present in individuals from other three groups, 1,146 loci that exists in two kinds of individuals and 2,806 loci present in only one (Table S6).

### Sex-linked markers validation

Thousands of putative loci were obtained through the above method and thus a stricter filtration with higher threshold was carried out to reduce the scope of sex-linked markers. Consequently, 154 putative loci were identified with high sequencing quality (read coverage > 15 ×), and 50 of them were chosen for validation (Table S7). Primers were supplied in Table S8. Among the fifty loci, eighteen loci with sex-limited occurrence were regarded as false positive after sanger sequencing and thirty of the rest with sex differences in heterozygosity were confirmed correct. The thirty SNPs are located on 28 different scaffolds of the Pacific oyster genome and their information was shown in Table S7. Next, they were examined using SNaPshot method in additional individuals from another population as described in the materials and methods section.

**Figure 1:**
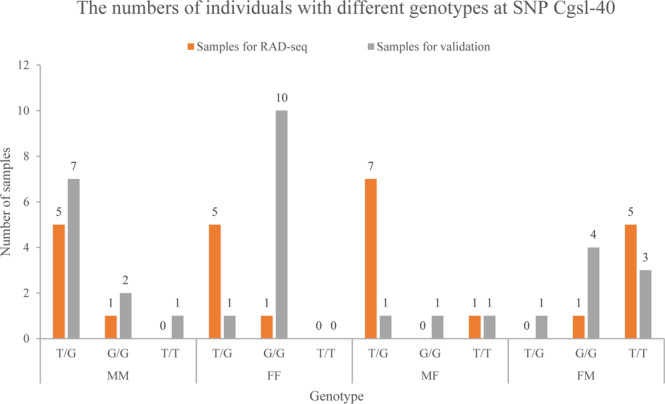
The numbers of individuals with different genotypes at SNP Cgsl-40. The horizontal axis represents genotype of individuals in the four phenotype groups. The orange and grey column represent samples for RAD-seq and validation, respectively. And the numbers of oysters are reported upon the column.

Twenty-nine of the thirty SNPs did not match sex-linked pattern in further validation as there is no significant difference of genotype frequency among different phenotype groups (*P* > 0.05; Supplementary Figure S1). One SNP Cgsl-40 genotype frequency showed obvious difference among validation groups (*P* < 0.05) but not consistent with RAD-seq result. The Cgsl-40 is located on the seventh exon of an uncharacterized gene LOC105342046 on scaffold NW_011936629.1 and the T to G transversion causes nonsynonymous mutation. On the loci, 83.33% individuals of FM group used for RAD-seq were T/T homozygotes, while most of others were T/G heterozygotes (Fig. 1). By contrast, in the SNaPshot analysis on additional individuals, 70.00% MM samples were T/G heterozygotes, 90.00% FF samples were G/G homozygotes, and there was no obvious distribution of dominant genotype in MF and FM samples (Fig. 1). The genotype frequency of SNP Cgsl-40 differed between additional MM and FF individuals (χ^2^ = 10.810, d.f. = 2, *P* = 0.004). When coming to MM and other phenotypic sex individuals, which means that FF, FM and MF individuals were assigned in one group, genotype frequency of Cgsl-40 was significantly different (χ^2^ = 10.288, d.f. = 2, *P* = 0.006), and similar pattern was discovered when FF and other samples were compared (χ^2^ = 9.757, d.f. = 2, *P* = 0.008).

## Discussion

The Pacific oyster *C. gigas* is a typical marine bivalve with occasional sex change and the sex conversion was thought to be responsible for varying sex ratios in accordance with age and/or size^14,25^. Until now, its sexual system has not been illuminated clearly. Some researchers considered the sexuality of *C. gigas* as protandric dioecy that the proportion of males is higher at the early stage, with increase in the proportion of females arising through sex reversal from male to female as the oyster gets older^14^. Direct sex observation by following the same individuals in this study does not support the protandry. Sex change in both directions was found in our study. Although the sex reversal rate from male to female (52.94%) was higher than that from female to male (20.83%), there is no difference in sex ratios of the investigated populations between one-year and two-year old as similar percentage of males (15.38%) and females (13.85%) had a sex change between the two seasons (Table 1). Female-biased sex ratios of oyster populations were reported^24^, which also challenged the protandry of *C. gigas*. Another sex ratio and sex reversal study claimed that the proportion of males was higher in the three-year-old class than in the two-year-old class^22^. This was viewed as the result of higher sex reversal from female to male than from male to female in two-year-old oysters^22^. From the above, our study provides evidence that the plasticity of sex in *C. giga*s was underestimated and the myth of protandry should be dispelled.

The complex sexuality of *C. gigas* makes it difficult to identify its type of sex determination and the question confusing researchers for a long time is whether sex chromosomes exist in this species. So far, cytogenetic analysis did not identify highly differentiated sex chromosomes in *C. gigas*^36^, but the presence of a sex chromosomal pair was not ruled out. Looking for potential sex chromosomes information in the Pacific oyster genome, samples for RAD-seq were divided into two groups according to their gender at the first maturation stage. This grouping was based on a hypothesis that a single predominant sex-determining region controls one-year-old oyster sexuality. In addition, the mitochondrial DNA data was excluded in the RAD-seq analysis as the Pacific oyster is a bivalve without doubly uniparental inheritance. Eight potential markers were identified through bioinformatic analysis, and one of the eight was chosen for validation in additional individuals but without sex-related pattern. This result suggests that the possibility for sex chromosomes in the Pacific oyster is quite low. Variable sex ratios in factorial and nested crosses (averaging 22.5 progeny for the 167 different families and approximately 3 times that for the half-sib families with a common male parent) were observed^14^, which also indicated the absence of sex chromosomes in *C. gigas*. Broods from species with strong chromosomal sex determination typically exhibit a narrow range of family sex ratios that do not divert substantially from 1:1^3,5,37^ and high variation in sex ratio across families is generally considered as a characteristic feature of polygenic sex determination^7^. Although a QTL mapping study of *C. gigas* that showed one linkage group acting like a sex chromosome was mentioned^25^, there is no more details that have been reported. Taken together, sex reversal, varying sex ratios among families and no universal sex-linked marker in one-year-old oysters suggest that *C. gigas* is unlikely to utilize chromosomal sex determination system.

As there is no proof of chromosomal sex determination system in *C. gigas*, another question occurred: does a single major gene control sex? Based on two-genotypes and three-genotypes genetic models for *C. gigas* sex determination^14,25^, individuals used for RAD-seq were assigned into three groups (MM, FF, and MF_FM) to identify sex-linked markers. Eighteen markers matched with two-genotypes model and three with three-genotypes model were selected for validation. But they were proved to be not sex-related in additional samples from another population. RAD-seq data were analyzed again when samples were grouped into four sets (MM, FF, MF and FM), mainly for additional FM or MF specific markers. Another twenty-eight markers beyond previous two genetic models were examined and one SNP Cgsl-40 showed genotype frequency difference among additional sample groups but inconsistent with the RAD-seq results. Taken together, there is no universal molecular marker linked with phenotypic sexes in distinct populations, which does not support a single dominant gene controlling the Pacific oyster sex. It should be noted that the four phenotypes (FF, MM, FM, and MF) may be biased for potential sex change in a part of FF and MM individuals after the first two breeding seasons, although sex change decreases for older oysters^34^. The negative effects of potential biased phenotypes on the absence of universal sex-linked markers were supposed to be reduced by lower criteria (80%) in our study compared with previous one (95%)^30^. And the absence of major sex-controlling gene was valid in the analyzed range of *C. gigas* genome as the read coverage rate was 12.03–23.44%. Moreover, these observations also suggest that genetic factors influencing sex may differ among different populations and/or environmental conditions in *C. gigas*. Similar population or strain dependent sex determination exists in zebrafish with polygenic sex determination system, as multiple independent genome-wide studies identified different sex-linked loci on the same and different chromosomes^38–40^. The result in our study points to the absence of evidence for a simple genetic sex determination system in *C. gigas* and leaves open the possibility that highly plastic genetic factors instead of a single major gene participate in the primary sex determination.

The mode of sexual reproduction of *C. gigas* is highly plastic and little is known about its sex determination system. Based on our result and previous researches, we propose a hypothesis that the phenotypic sex of *C. gigas* should be viewed as threshold quantitative trait that depends on the interactions of genetic factors and environmental conditions^6,7,10,24^. Moreover, there is a bound between the two directions of gonad differentiation. For a specific Pacific oyster individual, it has a bisexual potency in each reproduction cycle^41^. Most of the time, individual sexual fate is determined by activating one side of the bisexual potency. While, the drastic fluctuation of gene-environment interactions across the sex-determining threshold may result in rare hermaphrodites. Sex reversal should be regarded as the result of inverse gene-environment interactions between reproductive cycles. This is likely adaptive to the sessile life as the Pacific oyster cannot choose optimal circumstances by moving and it favors oyster population reproductive success under the given conditions that changes temporally^23^. What’s more, genetic diversity might benefit from sex reversal because it rises the chance of mating each other. As regards the genetic factors in such a complex system, we prefer polygenetic sex determination rather than a single major sex-determining gene because there is no evidence for single-locus models in our study. The polygenetic sex determination means that multiple sex-determining genes distribute across the oyster genome and the combination of their alleles play a major role in primary sex determination^5–7,42^. This polygenic sex determination hypothesis for *C. gigas* is tentative and detailed studies should be designed in future to provide more robust evidence.

In conclusion, the combination of sex observation by following the same individual at two maturation stages and RAD-seq technology did not identify consistent sex-linked marker among four phenotypic sexes individuals from distinct Pacific oyster populations. This result did not support sex chromosomes system or single-locus models for *C. gigas* primary sex determination. A sex determination hypothesis with multiple genetic factors is proposed based on our result and the associated studies. Information obtained in our study provides novel insight into sex determination mechanism in *C. gigas*.

## Supplementary information

Supplementary Information.

## Data Availability

Raw Illumina RAD-seq reads are available at the NCBI Short Read Archive (SRA, http://www.ncbi.nlm.nih.gov/sra/) in Bioproject PRJNA503968 under the accession number SRP167922.

## References

[1] Piferrer F (2013). Epigenetics of sex determination and gonadogenesis. Dev. Dyn..

[2] Manolakou P, Lavranos G, Angelopoulou R (2006). Molecular patterns of sex determination in the animal kingdom: a comparative study of the biology of reproduction. Reprod. Biol. Endocrinol..

[3] Baroiller JF, D’Cotta H, Bezault E, Wessels S, Hoerstgen-Schwark G (2009). Tilapia sex determination: where temperature and genetics meet. Comp. Biochem. Physiol., Part A: Mol. Integr. Physiol..

[4] Wilson MA, Makova KD (2009). Genomic analyses of sex chromosome evolution. Annu. Rev. Genomics Hum. Genet..

[5] Liew WC (2012). Polygenic sex determination system in zebrafish. PloS One.

[6] Bull J, Vogt RC, Bulmer M (1982). Heritability of sex ratio in turtles with environmental sex determination. Evolution.

[7] Bateman AW, Anholt BR (2017). Maintenance of polygenic sex determination in a fluctuating environment: an individual-based model. J. Evol. Biol..

[8] Modi WS, Crews D (2005). Sex chromosomes and sex determination in reptiles: Commentary. Curr. Opin. Genet. Dev..

[9] Grossen C, Neuenschwander S, Perrin N (2011). Temperature-dependent turnovers in sex-determination mechanisms: a quantitative model. Evolution.

[10] Santerre C (2013). Oyster sex determination is influenced by temperature—first clues in spat during first gonadic differentiation and gametogenesis. Comp. Biochem. Physiol., Part A: Mol. Integr. Physiol..

[11] Liew WC, Orban L (2014). Zebrafish sex: a complicated affair. Briefings Funct. Genomics.

[12] Palaiokostas C (2015). A new SNP-based vision of the genetics of sex determination in European sea bass (*Dicentrarchus labrax*). Genet., Sel., Evol..

[13] Coe WR (1943). Sexual differentiation in mollusks. I. Pelecypods. Q. Rev. Biol..

[14] Guo X, Hedgecock D, Hershberger WK, Cooper K, Allen SK (1998). Genetic determinants of protandric sex in the Pacific oyster, *Crassostrea gigas* Thunberg. Evolution.

[15] Breton, S., Capt, C., Guerra, D., & Stewart, D. Sex-determining mechanisms in bivalves in Transitions Between Sexual Systems (ed. Leonard, J.L.) 165-192 (Springer, 2018).

[16] Guo X, Allen SK (1994). Sex determination and polyploid gigantism in the dwarf surfclam (*Mulinia lateralis* Say). Genetics.

[17] Coe WR (1932). Sexual phases in the American oyster (*Ostrea Virginica*). Biol. Bull..

[18] Chávez-Villalba J (2011). Determination of gender in the pearl oyster *Pinctada margaritifera*. J. Shellfish Res..

[19] Coe WR (1936). Environment and sex in the oviparous oyster *Ostrea virginica*. Biol. Bull..

[20] Haley LE (1977). Sex determination in the American oyster. J. Hered..

[21] Haley LE (1979). Genetics of sex determination in the American oyster. Proc Natl Shellfishries Association.

[22] Park JJ (2012). Sex ratio and sex reversal in two-year-old class of oyster, *Crassostrea gigas* (Bivalvia: Ostreidae). Dev. Reprod..

[23] Yasuoka N, Yusa Y (2016). Effects of size and gregariousness on individual sex in a natural population of the Pacific oyster *Crassostrea gigas*. J. Mollus. Stud..

[24] Lango-Reynoso F, Chavez-Villaba J, Le Pennec M (2006). Reproductive patterns of the Pacific oyster *Crassostrea gigas* in France. Invertebr. Reprod. Dev..

[25] Hedrick PW, Hedgecock D (2010). Sex determination: genetic models for oysters. J. Hered..

[26] Li L, Guo X (2004). AFLP-based genetic linkage maps of the Pacific oyster *Crassostrea gigas* Thunberg. Mar. Biotechnol..

[27] Zhang N, Xu F, Guo X (2014). Genomic analysis of the Pacific oyster (*Crassostrea gigas*) reveals possible conservation of vertebrate sex determination in a mollusc. G3: Genes, Genomes, Genet..

[28] Yue C, Li Q, Yu H (2018). Gonad transcriptome analysis of the Pacific oyster *Crassostrea gigas* identifies potential genes regulating the sex determination and differentiation process. Mar. Biotechnol..

[29] Santerre C, Sourdaine P, Martinez A-S (2012). Expression of a natural antisense transcript of Cg-Foxl2 during the gonadic differentiation of the oyster *Crassostrea gigas*: first demonstration in the gonads of a lophotrochozoa species. Sex. Dev..

[30] Brelsford A, Lavanchy G, Sermier R, Rausch A, Perrin N (2017). Identifying homomorphic sex chromosomes from wild-caught adults with limited genomic resources. Mol. Ecol. Resour..

[31] Gamble T, Zarkower D (2014). Identification of sex-specific molecular markers using restriction site-associated DNA sequencing. Mol. Ecol. Resour..

[32] Gamble T (2015). Restriction site-associated DNA sequencing (RAD-seq) reveals an extraordinary number of transitions among gecko sex-determining systems. Mol. Biol. Evol..

[33] Liu H (2018). Sex-specific markers developed by next-generation sequencing confirmed an XX/XY sex determination system in bighead carp (*Hypophthalmichehys nobilis*) and silver carp (*Hypophthalmichthys molitrix*). DNA Res..

[34] Broquard C, Martinez AS, Maurouard E, Lamy JB, Dégremont L (2020). Sex determination in the oyster *Crassostrea gigas*-A large longitudinal study of population sex ratios and individual sex changes. Aquaculture.

[35] Namba K (1995). Persistent relaxation of the adductor muscle of oyster *Crassostrea gigas* induced by magnesium ion. Fish. Sci..

[36] Leitão A, Chaves R, Santos S, Guedes-Pinto H, Boudry P (2007). Interspecific hybridization in oysters: restriction enzyme digestion chromosome banding confirms *Crassostrea angulata*×*Crassostrea gigas* F1 hybrids. J. Exp. Mar. Biol. Ecol..

[37] Magerhans A, Müller-Belecke A, Hörstgen-Schwark G (2009). Effect of rearing temperatures post hatching on sex ratios of rainbow trout (*Oncorhynchus mykiss*) populations. Aquaculture.

[38] Bradley KM (2011). An SNP-based linkage map for zebrafish reveals sex determination loci. G3: Genes, Genomes, Genet..

[39] Howe K (2013). The zebrafish reference genome sequence and its relationship to the human genome. Nature.

[40] Nagabhushana A, Mishra RK (2016). Finding clues to the riddle of sex determination in zebrafish. J. Biosci..

[41] Kosswig C (1964). Polygenic sex determination. Cell. Mol. Life Sci..

[42] Bull J (1985). Sex determining mechanisms: an evolutionary perspective. Experientia.

