# Supplementary material for: Restriction site-associated DNA sequencing (RAD-seq) analysis in Pacific oyster Crassostrea gigas based on observation of individual sex changes
Source: Sci Rep. 2020 Jun 18;10:9873. doi: 10.1038/s41598-020-67007-4 (PMC7303127; doi:10.1038/s41598-020-67007-4)
Supplement: Supplementary file 1 — Supplementary Information. [file 41598_2020_67007_MOESM1_ESM.doc]

**Restriction site-associated DNA sequencing (RAD-seq) analysis in Pacific oyster *Crassostrea gigas* based on observation of individual sex changes**

Chenyang Yue · Qi Li · Hong Yu · Shikai Liu · Lingfeng Kong

**Table S**1 Summary of RAD-seq results

| **Samples** | **Raw reads** | **Clean reads** | **Q30** | **Align reads** | **Mean depth** | **Coverage rate** |
| --- | --- | --- | --- | --- | --- | --- |
| FF1 | 11,436,080 | 10,438,086 | 95.63% | 7160184 | 7.50X | 14.15% |
| FF2 | 8,774,158 | 8,593,138 | 94.23% | 7717319 | 7.65X | 19.26% |
| FF3 | 10,533,134 | 10,313,668 | 94.40% | 9077429 | 8.38X | 20.45% |
| FF4 | 11,402,118 | 11,184,208 | 94.63% | 10032311 | 9.29X | 20.31% |
| FF5 | 9,726,792 | 9,511,830 | 94.11% | 8539298 | 8.05X | 20.28% |
| FF6 | 10,956,946 | 10,733,382 | 94.97% | 9647669 | 9.04X | 20.40% |
| MM1 | 11,928,714 | 10,426,940 | 95.63% | 7170525 | 8.61X | 12.45% |
| MM2 | 12,485,482 | 12,205,232 | 94.23% | 10867070 | 9.1X | 22.43% |
| MM3 | 10,063,598 | 9,775,964 | 94.40% | 8715374 | 7.04X | 23.44% |
| MM4 | 9,741,610 | 9,547,218 | 94.63% | 8564479 | 8.5X | 19.25% |
| MM5 | 10,812,674 | 10,545,972 | 94.11% | 9468923 | 7.99X | 22.49% |
| MM6 | 11,028,298 | 10,788,946 | 94.97% | 9719004 | 8.47X | 22.07% |
| MF1 | 11,794,192 | 10,295,528 | 95.62% | 7012752 | 8.19X | 12.74% |
| MF2 | 12,083,870 | 10,254,448 | 95.87% | 7033047 | 8.5X | 12.41% |
| MF3 | 12,366,536 | 12,366,536 | 95.44% | 7101625 | 7.77X | 13.62% |
| MF4 | 12,366,536 | 10,241,180 | 95.68% | 7011824 | 8.33X | 12.48% |
| MF5 | 11,118,710 | 10,424,048 | 95.45% | 7145954 | 8.47X | 12.54% |
| MF6 | 9,822,496 | 9,512,180 | 92.89% | 8513422 | 6.9X | 23.43% |
| MF7 | 11,725,930 | 11,460,290 | 94.20% | 10259068 | 9.63X | 20.40% |
| MF8 | 9,966,260 | 9,700,248 | 93.82% | 8715154 | 7.37X | 22.46% |
| FM1 | 10,732,910 | 10,095,538 | 95.80% | 6,956,133 | 8.47X | 12.26% |
| FM2 | 11,993,098 | 11,619,348 | 95.84% | 7,999,311 | 9.70X | 12.22% |
| FM3 | 10,999,746 | 10,439,010 | 95.87% | 7,239,469 | 8.84X | 12.24% |
| FM4 | 11,154,120 | 10,402,660 | 95.82% | 7,195,098 | 8.93X | 12.03% |
| FM5 | 12,378,390 | 11,839,390 | 95.79% | 8,143,851 | 9.67X | 12.48% |
| FM6 | 9,277,174 | 9,068,568 | 94.36% | 8135605 | 7.69X | 20.12% |

**Table S2 Structure information of variants called by GATK software.**

| **Structure type** | **Number of variants** | **Percentage** |
| --- | --- | --- |
| Exon | 634,226 | 7.04% |
| Intergenic region | 3,364,202 | 37.34% |
| Intron | 3,613,657 | 40.11% |
| NcRNA exon | 49,990 | 0.55% |
| NcRNA intron | 164,278 | 1.82% |
| NcRNA splicing region | 232 | 0.00% |
| NcRNA UTR | 1,138 | 0.01% |
| Splicing region | 2,312 | 0.03% |
| Upstream or downstream | 870,198 | 9.66% |
| UTR | 309,496 | 3.44% |
| Null | 38 | 0.00% |

**Table S3 Function information of variants called by GATK software**.

| **Function type** | **Number of variants** | **Percentage** |
| --- | --- | --- |
| Unknown | 8,376,329 | 92.99% |
| Frameshift deletion | 2,734 | 0.03% |
| Frameshift insertion | 1,608 | 0.02% |
| Nonframeshift deletion | 2,156 | 0.02% |
| Nonframeshift insertion | 1,492 | 0.02% |
| Nonsynonymous SNV | 263,361 | 2.92% |
| Synonymous SNV | 354,703 | 3.94% |
| Stopgain | 4,697 | 0.05% |
| Stoploss | 475 | 0.01% |

**Table S4 Summary of loci identified by the first** grouping.

| **MM_MF group** | **FF_FM group** | **Loci number** |
| --- | --- | --- |
| 0|0 | 1|1 | 0 |
| 1|1 | 0|0 | 0 |
| 0|1 | 0|0 | 0 |
| 0|1 | 1|1 | 0 |
| 0|0 | 0|1 | 1 |
| 1|1 | 0|1 | 0 |
| .|. | 0|0 | 0 |
| .|. | 1|1 | 2 |
| .|. | 0|1 | 0 |
| 0|0 | .|. | 0 |
| 1|1 | .|. | 5 |
| 0|1 | .|. | 0 |

0|0 and 1|1: homozygote; 0|1: heterozygote; .|.: deletant.

**Table S5 Summary of loci identified by the second grouping.**

| **MM group** | **FF group** | **MF_FM group** | **Loci number** |
| --- | --- | --- | --- |
| .|. | .|. | 0|0 | 1 |
| .|. | .|. | 0|1 | 0 |
| .|. | .|. | 1|1 | 10 |
| .|. | 0|0 | 0|0 | 5 |
| .|. | 0|0 | 0|1 | 0 |
| .|. | 0|0 | 1|1 | 0 |
| .|. | 0|0 | .|. | 701 |
| .|. | 0|1 | 0|0 | 0 |
| .|. | 0|1 | 0|1 | 0 |
| .|. | 0|1 | 1|1 | 0 |
| .|. | 0|1 | .|. | 0 |
| .|. | 1|1 | 0|0 | 0 |
| .|. | 1|1 | 0|1 | 0 |
| .|. | 1|1 | 1|1 | 30 |
| .|. | 1|1 | .|. | 529 |
| 0|0 | .|. | 0|0 | 5 |
| 0|0 | .|. | 0|1 | 0 |
| 0|0 | .|. | 1|1 | 0 |
| 0|0 | .|. | .|. | 1830 |
| 0|0 | 0|0 | 0|1 | 2 |
| 0|0 | 0|0 | 1|1 | 0 |
| 0|0 | 0|0 | .|. | 1875 |
| 0|0 | 0|1 | 0|0 | 43 |
| 0|0 | 0|1 | 0|1 | 5 |
| 0|0 | 0|1 | 1|1 | 0 |
| 0|0 | 0|1 | .|. | 0 |
| 0|0 | 1|1 | 0|0 | 0 |
| 0|0 | 1|1 | 0|1 | 0 |
| 0|0 | 1|1 | 1|1 | 0 |
| 0|0 | 1|1 | .|. | 3 |
| 0|1 | .|. | 0|0 | 0 |
| 0|1 | .|. | 0|1 | 0 |
| 0|1 | .|. | 1|1 | 0 |
| 0|1 | .|. | .|. | 3 |
| 0|1 | 0|0 | 0|0 | 36 |
| 0|1 | 0|0 | 0|1 | 0 |
| 0|1 | 0|0 | 1|1 | 0 |
| 0|1 | 0|0 | .|. | 2 |
| 0|1 | 0|1 | 0|0 | 3 |
| 0|1 | 0|1 | 1|1 | 4 |
| 0|1 | 0|1 | .|. | 3 |
| 0|1 | 1|1 | 0|0 | 0 |
| 0|1 | 1|1 | 0|1 | 1 |
| 0|1 | 1|1 | 1|1 | 9 |
| 0|1 | 1|1 | .|. | 0 |
| 1|1 | .|. | 0|0 | 0 |
| 1|1 | .|. | 0|1 | 0 |
| 1|1 | .|. | 1|1 | 28 |
| 1|1 | .|. | .|. | 1021 |
| 1|1 | 0|0 | 0|0 | 0 |
| 1|1 | 0|0 | 0|1 | 0 |
| 1|1 | 0|0 | 1|1 | 0 |
| 1|1 | 0|0 | .|. | 0 |
| 1|1 | 0|1 | 0|0 | 0 |
| 1|1 | 0|1 | 0|1 | 2 |
| 1|1 | 0|1 | 1|1 | 4 |
| 1|1 | 0|1 | .|. | 0 |
| 1|1 | 1|1 | 0|0 | 0 |
| 1|1 | 1|1 | 0|1 | 0 |
| 1|1 | 1|1 | .|. | 763 |

0|0 and 1|1: homozygote; 0|1: heterozygote; .|.; deletant.

**Table S6 Summary of loci identified by the third grouping.**

| **MM group** | **MF group** | **FF group** | **FM group** | **Loci number** |
| --- | --- | --- | --- | --- |
| 0|0 | 1|1 | 1|1 | 1|1 | 0 |
| 1|1 | 0|0 | 0|0 | 0|0 | 0 |
| 1|1 | 0|0 | 1|1 | 1|1 | 0 |
| 0|0 | 1|1 | 0|0 | 0|0 | 0 |
| 1|1 | 1|1 | 0|0 | 1|1 | 0 |
| 0|0 | 0|0 | 1|1 | 0|0 | 0 |
| 1|1 | 1|1 | 1|1 | 0|0 | 0 |
| 0|0 | 0|0 | 0|0 | 1|1 | 0 |
| 0|1 | 0|0 | 0|0 | 0|0 | 21 |
| 0|1 | 0|0 | 0|0 | 1|1 | 0 |
| 0|1 | 0|0 | 1|1 | 0|0 | 0 |
| 0|1 | 0|0 | 1|1 | 1|1 | 0 |
| 0|1 | 1|1 | 0|0 | 0|0 | 0 |
| 0|1 | 1|1 | 0|0 | 1|1 | 0 |
| 0|1 | 1|1 | 1|1 | 0|0 | 0 |
| 0|1 | 1|1 | 1|1 | 1|1 | 5 |
| 0|0 | 0|1 | 0|0 | 0|0 | 8 |
| 0|0 | 0|1 | 0|0 | 1|1 | 0 |
| 0|0 | 0|1 | 1|1 | 0|0 | 0 |
| 0|0 | 0|1 | 1|1 | 1|1 | 0 |
| 1|1 | 0|1 | 0|0 | 0|0 | 0 |
| 1|1 | 0|1 | 0|0 | 1|1 | 0 |
| 1|1 | 0|1 | 1|1 | 0|0 | 0 |
| 1|1 | 0|1 | 1|1 | 1|1 | 2 |
| 0|0 | 0|0 | 0|1 | 0|0 | 30 |
| 0|0 | 0|0 | 0|1 | 1|1 | 0 |
| 0|0 | 1|1 | 0|1 | 0|0 | 0 |
| 0|0 | 1|1 | 0|1 | 1|1 | 0 |
| 1|1 | 0|0 | 0|1 | 0|0 | 0 |
| 1|1 | 0|0 | 0|1 | 1|1 | 0 |
| 1|1 | 1|1 | 0|1 | 0|0 | 0 |
| 1|1 | 1|1 | 0|1 | 1|1 | 2 |
| 0|0 | 0|0 | 0|0 | 0|1 | 15 |
| 0|0 | 0|0 | 1|1 | 0|1 | 0 |
| 0|0 | 1|1 | 0|0 | 0|1 | 0 |
| 0|0 | 1|1 | 1|1 | 0|1 | 0 |
| 1|1 | 0|0 | 0|0 | 0|1 | 0 |
| 1|1 | 0|0 | 1|1 | 0|1 | 0 |
| 1|1 | 1|1 | 0|0 | 0|1 | 0 |
| 1|1 | 1|1 | 1|1 | 0|1 | 2 |
| 0|1 | 0|1 | 0|0 | 0|0 | 0 |
| 0|1 | 0|1 | 1|1 | 1|1 | 0 |
| 0|1 | 0|1 | 0|0 | 1|1 | 0 |
| 0|1 | 0|1 | 1|1 | 0|0 | 0 |
| 0|1 | 0|0 | 0|1 | 0|0 | 1 |
| 0|1 | 1|1 | 0|1 | 1|1 | 3 |
| 0|1 | 0|0 | 0|1 | 1|1 | 0 |
| 0|1 | 1|1 | 0|1 | 0|0 | 0 |
| 0|1 | 0|0 | 0|0 | 0|1 | 0 |
| 0|1 | 1|1 | 1|1 | 0|1 | 0 |
| 0|1 | 0|0 | 1|1 | 0|1 | 0 |
| 0|1 | 1|1 | 0|0 | 0|1 | 0 |
| 0|0 | 0|1 | 0|1 | 0|0 | 1 |
| 1|1 | 0|1 | 0|1 | 1|1 | 0 |
| 0|0 | 0|1 | 0|1 | 1|1 | 0 |
| 1|1 | 0|1 | 0|1 | 0|0 | 0 |
| 0|0 | 0|1 | 0|0 | 0|1 | 0 |
| 1|1 | 0|1 | 1|1 | 0|1 | 0 |
| 0|0 | 0|1 | 1|1 | 0|1 | 0 |
| 1|1 | 0|1 | 0|0 | 0|1 | 0 |
| 0|0 | 0|0 | 0|1 | 0|1 | 0 |
| 1|1 | 1|1 | 0|1 | 0|1 | 0 |
| 0|0 | 1|1 | 0|1 | 0|1 | 0 |
| 1|1 | 0|0 | 0|1 | 0|1 | 0 |
| 0|1 | 0|1 | 0|1 | 0|0 | 1 |
| 0|1 | 0|1 | 0|1 | 1|1 | 2 |
| 0|1 | 0|1 | 0|0 | 0|1 | 0 |
| 0|1 | 0|1 | 1|1 | 0|1 | 1 |
| 0|1 | 0|0 | 0|1 | 0|1 | 2 |
| 0|1 | 1|1 | 0|1 | 0|1 | 0 |
| 0|0 | 0|1 | 0|1 | 0|1 | 3 |
| 1|1 | 0|1 | 0|1 | 0|1 | 1 |
| .|. | 0|0 | 0|0 | 0|0 | 0 |
| .|. | 0|0 | 0|0 | 1|1 | 0 |
| .|. | 0|0 | 0|0 | 0|1 | 0 |
| .|. | 0|0 | 1|1 | 0|0 | 0 |
| .|. | 0|0 | 1|1 | 1|1 | 0 |
| .|. | 0|0 | 1|1 | 0|1 | 0 |
| .|. | 0|0 | 0|1 | 0|0 | 0 |
| .|. | 0|0 | 0|1 | 1|1 | 0 |
| .|. | 0|0 | 0|1 | 0|1 | 0 |
| .|. | 1|1 | 0|0 | 0|0 | 0 |
| .|. | 1|1 | 0|0 | 1|1 | 0 |
| .|. | 1|1 | 0|0 | 0|1 | 0 |
| .|. | 1|1 | 1|1 | 0|0 | 0 |
| .|. | 1|1 | 1|1 | 1|1 | 24 |
| .|. | 1|1 | 1|1 | 0|1 | 0 |
| .|. | 1|1 | 0|1 | 0|0 | 0 |
| .|. | 1|1 | 0|1 | 1|1 | 0 |
| .|. | 1|1 | 0|1 | 0|1 | 0 |
| .|. | 0|1 | 0|0 | 0|0 | 0 |
| .|. | 0|1 | 0|0 | 1|1 | 0 |
| .|. | 0|1 | 0|0 | 0|1 | 0 |
| .|. | 0|1 | 1|1 | 0|0 | 0 |
| .|. | 0|1 | 1|1 | 1|1 | 0 |
| .|. | 0|1 | 1|1 | 0|1 | 0 |
| .|. | 0|1 | 0|1 | 0|0 | 0 |
| .|. | 0|1 | 0|1 | 1|1 | 0 |
| .|. | 0|1 | 0|1 | 0|1 | 0 |
| 0|0 | .|. | 0|0 | 0|0 | 0 |
| 0|0 | .|. | 0|0 | 1|1 | 0 |
| 0|0 | .|. | 0|0 | 0|1 | 0 |
| 0|0 | .|. | 1|1 | 0|0 | 0 |
| 0|0 | .|. | 1|1 | 1|1 | 0 |
| 0|0 | .|. | 1|1 | 0|1 | 0 |
| 0|0 | .|. | 0|1 | 0|0 | 0 |
| 0|0 | .|. | 0|1 | 1|1 | 0 |
| 0|0 | .|. | 0|1 | 0|1 | 0 |
| 1|1 | .|. | 0|0 | 0|0 | 0 |
| 1|1 | .|. | 0|0 | 1|1 | 0 |
| 1|1 | .|. | 0|0 | 0|1 | 0 |
| 1|1 | .|. | 1|1 | 0|0 | 0 |
| 1|1 | .|. | 1|1 | 1|1 | 3 |
| 1|1 | .|. | 1|1 | 0|1 | 0 |
| 1|1 | .|. | 0|1 | 0|0 | 0 |
| 1|1 | .|. | 0|1 | 1|1 | 0 |
| 1|1 | .|. | 0|1 | 0|1 | 0 |
| 0|1 | .|. | 0|0 | 0|0 | 0 |
| 0|1 | .|. | 0|0 | 1|1 | 0 |
| 0|1 | .|. | 0|0 | 0|1 | 0 |
| 0|1 | .|. | 1|1 | 0|0 | 0 |
| 0|1 | .|. | 1|1 | 1|1 | 0 |
| 0|1 | .|. | 1|1 | 0|1 | 0 |
| 0|1 | .|. | 0|1 | 0|0 | 0 |
| 0|1 | .|. | 0|1 | 1|1 | 0 |
| 0|1 | .|. | 0|1 | 0|1 | 0 |
| 0|0 | 0|0 | .|. | 0|0 | 2 |
| 0|0 | 0|0 | .|. | 1|1 | 0 |
| 0|0 | 0|0 | .|. | 0|1 | 0 |
| 0|0 | 1|1 | .|. | 0|0 | 0 |
| 0|0 | 1|1 | .|. | 1|1 | 0 |
| 0|0 | 1|1 | .|. | 0|1 | 0 |
| 0|0 | 0|1 | .|. | 0|0 | 0 |
| 0|0 | 0|1 | .|. | 1|1 | 0 |
| 0|0 | 0|1 | .|. | 0|1 | 0 |
| 1|1 | 0|0 | .|. | 0|0 | 0 |
| 1|1 | 0|0 | .|. | 1|1 | 0 |
| 1|1 | 0|0 | .|. | 0|1 | 0 |
| 1|1 | 1|1 | .|. | 0|0 | 0 |
| 1|1 | 1|1 | .|. | 1|1 | 20 |
| 1|1 | 1|1 | .|. | 0|1 | 0 |
| 1|1 | 0|1 | .|. | 0|0 | 0 |
| 1|1 | 0|1 | .|. | 1|1 | 0 |
| 1|1 | 0|1 | .|. | 0|1 | 0 |
| 0|1 | 0|0 | .|. | 0|0 | 0 |
| 0|1 | 0|0 | .|. | 1|1 | 0 |
| 0|1 | 0|0 | .|. | 0|1 | 0 |
| 0|1 | 1|1 | .|. | 0|0 | 0 |
| 0|1 | 1|1 | .|. | 1|1 | 0 |
| 0|1 | 1|1 | .|. | 0|1 | 0 |
| 0|1 | 0|1 | .|. | 0|0 | 0 |
| 0|1 | 0|1 | .|. | 1|1 | 0 |
| 0|1 | 0|1 | .|. | 0|1 | 0 |
| 0|0 | 0|0 | 0|0 | .|. | 5 |
| 0|0 | 0|0 | 1|1 | .|. | 0 |
| 0|0 | 0|0 | 0|1 | .|. | 0 |
| 0|0 | 1|1 | 0|0 | .|. | 0 |
| 0|0 | 1|1 | 1|1 | .|. | 0 |
| 0|0 | 1|1 | 0|1 | .|. | 0 |
| 0|0 | 0|1 | 0|0 | .|. | 0 |
| 0|0 | 0|1 | 1|1 | .|. | 0 |
| 0|0 | 0|1 | 0|1 | .|. | 0 |
| 1|1 | 0|0 | 0|0 | .|. | 0 |
| 1|1 | 0|0 | 1|1 | .|. | 0 |
| 1|1 | 0|0 | 0|1 | .|. | 0 |
| 1|1 | 1|1 | 0|0 | .|. | 0 |
| 1|1 | 1|1 | 1|1 | .|. | 84 |
| 1|1 | 1|1 | 0|1 | .|. | 0 |
| 1|1 | 0|1 | 0|0 | .|. | 0 |
| 1|1 | 0|1 | 1|1 | .|. | 0 |
| 1|1 | 0|1 | 0|1 | .|. | 0 |
| 0|1 | 0|0 | 0|0 | .|. | 0 |
| 0|1 | 0|0 | 1|1 | .|. | 0 |
| 0|1 | 0|0 | 0|1 | .|. | 0 |
| 0|1 | 1|1 | 0|0 | .|. | 0 |
| 0|1 | 1|1 | 1|1 | .|. | 0 |
| 0|1 | 1|1 | 0|1 | .|. | 0 |
| 0|1 | 0|1 | 0|0 | .|. | 0 |
| 0|1 | 0|1 | 1|1 | .|. | 0 |
| 0|1 | 0|1 | 0|1 | .|. | 0 |
| .|. | .|. | 0|0 | 0|0 | 0 |
| .|. | .|. | 0|0 | 1|1 | 0 |
| .|. | .|. | 0|0 | 0|1 | 0 |
| .|. | .|. | 1|1 | 0|0 | 0 |
| .|. | .|. | 1|1 | 1|1 | 1 |
| .|. | .|. | 1|1 | 0|1 | 0 |
| .|. | .|. | 0|1 | 0|0 | 0 |
| .|. | .|. | 0|1 | 1|1 | 0 |
| .|. | .|. | 0|1 | 0|1 | 0 |
| .|. | 0|0 | .|. | 0|0 | 0 |
| .|. | 0|0 | .|. | 1|1 | 0 |
| .|. | 0|0 | .|. | 0|1 | 0 |
| .|. | 1|1 | .|. | 0|0 | 0 |
| .|. | 1|1 | .|. | 1|1 | 8 |
| .|. | 1|1 | .|. | 0|1 | 0 |
| .|. | 0|1 | .|. | 0|0 | 0 |
| .|. | 0|1 | .|. | 1|1 | 0 |
| .|. | 0|1 | .|. | 0|1 | 0 |
| .|. | 0|0 | 0|0 | .|. | 0 |
| .|. | 0|0 | 1|1 | .|. | 0 |
| .|. | 0|0 | 0|1 | .|. | 0 |
| .|. | 1|1 | 0|0 | .|. | 0 |
| .|. | 1|1 | 1|1 | .|. | 2 |
| .|. | 1|1 | 0|1 | .|. | 0 |
| .|. | 0|1 | 0|0 | .|. | 0 |
| .|. | 0|1 | 1|1 | .|. | 0 |
| .|. | 0|1 | 0|1 | .|. | 0 |
| 0|0 | .|. | .|. | 0|0 | 0 |
| 0|0 | .|. | .|. | 1|1 | 0 |
| 0|0 | .|. | .|. | 0|1 | 0 |
| 1|1 | .|. | .|. | 0|0 | 1 |
| 1|1 | .|. | .|. | 1|1 | 0 |
| 1|1 | .|. | .|. | 0|1 | 0 |
| 0|1 | .|. | .|. | 0|0 | 0 |
| 0|1 | .|. | .|. | 1|1 | 0 |
| 0|1 | .|. | .|. | 0|1 | 0 |
| 0|0 | .|. | 0|0 | .|. | 803 |
| 0|0 | .|. | 1|1 | .|. | 3 |
| 0|0 | .|. | 0|1 | .|. | 0 |
| 1|1 | .|. | 0|0 | .|. | 0 |
| 1|1 | .|. | 1|1 | .|. | 324 |
| 1|1 | .|. | 0|1 | .|. | 0 |
| 0|1 | .|. | 0|0 | .|. | 1 |
| 0|1 | .|. | 1|1 | .|. | 0 |
| 0|1 | .|. | 0|1 | .|. | 3 |
| 0|0 | 0|0 | .|. | .|. | 0 |
| 0|0 | 1|1 | .|. | .|. | 0 |
| 0|0 | 0|1 | .|. | .|. | 0 |
| 1|1 | 0|0 | .|. | .|. | 0 |
| 1|1 | 1|1 | .|. | .|. | 0 |
| 1|1 | 0|1 | .|. | .|. | 0 |
| 0|1 | 0|0 | .|. | .|. | 0 |
| 0|1 | 1|1 | .|. | .|. | 0 |
| 0|1 | 0|1 | .|. | .|. | 0 |
| .|. | .|. | .|. | 0|0 | 177 |
| .|. | .|. | .|. | 1|1 | 83 |
| .|. | .|. | .|. | 0|1 | 0 |
| .|. | .|. | 0|0 | .|. | 437 |
| .|. | .|. | 1|1 | .|. | 380 |
| .|. | .|. | 0|1 | .|. | 0 |
| .|. | 0|0 | .|. | .|. | 0 |
| .|. | 1|1 | .|. | .|. | 4 |
| .|. | 0|1 | .|. | .|. | 0 |
| 0|0 | .|. | .|. | .|. | 1019 |
| 1|1 | .|. | .|. | .|. | 706 |
| 0|1 | .|. | .|. | .|. | 0 |

0|0 and 1|1: homozygote; 0|1: heterozygote; .|.; deletant.

**Table S**7 Primers used to amplify potential sex-linked markers.

| **Primer name** | **Primer sequence (5' to 3')** | **Amplicon lengths (bp)** | **Tm (℃)** |
| --- | --- | --- | --- |
| Cgsl-1F | TCTCAAGACTCGTTATGCT | 146 | 52 |
| Cgsl-1R | CAAATTGCGGTAGGGAAA |  |  |
| Cgsl-2F | ATGCTCTTTTGGAAGGGTGG | 335 | 56 |
| Cgsl-2R | TCAAGCAGAGGCCACATGTC |  |  |
| Cgsl-3F | AATACGAACTATGCTTCTGAG | 211 | 52 |
| Cgsl-3R | CCGACAATGATTCTCTTCC |  |  |
| Cgsl-4F | GTGAAGAGTGACCGCTAT | 199 | 52 |
| Cgsl-4R | TACTTGACCTGTGGAACC |  |  |
| Cgsl-5F | TCACCGCGACATAAATTACGAACAA | 438 | 60 |
| Cgsl-5R | TCAAGAGCTGGAAAGACTCCGATTT |  |  |
| Cgsl-6F | GATGAAGGACTATTGAGGAAG | 199 | 52 |
| Cgsl-6R | GAACTGCTGAACAATCACT |  |  |
| Cgsl-7F | CGGCTAAGTCACTGTGTA | 222 | 52 |
| Cgsl-7R | GCAATATACTGTCTTCCTGTT |  |  |
| Cgsl-8F | CGCTACATGGCACCAGAATTGTTG | 683 | 60 |
| Cgsl-8R | AGTAACTGTTCACAGCCTCACATCA |  |  |
| Cgsl-9F | ATGAGGTGGCAGGGAATATG | 335 | 54 |
| Cgsl-9R | GGAGGCAGAAGAAAGAAACG |  |  |
| Cgsl-10F | TTGGTCAGGTGAACTACT | 142 | 52 |
| Cgsl-10R | TTGGTGGACTTGATGAAC |  |  |
| Cgsl-11F | CAAACACTCTGGCTACAT | 147 | 52 |
| Cgsl-11R | CGGAAACAACTGGTCTAC |  |  |
| Cgsl-12F | CAAATCACATCCCTCTTATCATA | 134 | 52 |
| Cgsl-12F | CCAATGCTTGCCAATAATG |  |  |
| Cgsl-13F | AGGGCACTTCTCTATCAGCACAAAC | 528 | 60 |
| Cgsl-13R | ACAGCGTAATTATCGTCCTCGTTCA |  |  |
| Cgsl-14F | ACAGTGAGTCTACCTTTGCACT | 104 | 56 |
| Cgsl-14R | ACAGCGTCTGCCATCACTAC |  |  |
| Cgsl-15F | ATGAGGTTCCGCTATATCTG | 740 | 52 |
| Cgsl-15R | AATGTCTATGTCTGCTCTCC |  |  |
| Cgsl-16F | AAGGCGGCTATTGTTGAA | 287 | 56 |
| Cgsl-16R | GCATAGAATAGAGAAGACTTCC |  |  |
| Cgsl-17F | TGCGGCCGATTTCTTGAAT | 145 | 56 |
| Cgsl-17R | GAGCCACCATCTACACCACAC |  |  |
| Cgsl-18F | TTGGAAATGATCTATGCTGAAA | 197 |  |
| Cgsl-18R | ACTGCGTCAACTATACTCTT |  |  |
| Cgsl-19F | GCAAGGAAAAGGAATAACATT | 196 |  |
| Cgsl-19R | AAGAACAAGTAGGAAATCGTAT |  |  |
| Cgsl-20F | CCAGACTAAGAATGCGTCTATTG | 750 | 56 |
| Cgsl-20R | CATAACCAACTTCGTCCATCAG |  |  |
| Cgsl-21&22F | GGAGGCTCATAGTGTTAATACAG | 504 | 60 |
| Cgsl-21&22R | CATTAGGGATGTAGCAAAGAGAC |  |  |
| Cgsl-23F | TGGAAGGAATATGCTGTTGA | 433 | 58 |
| Cgsl-23R | AACCATTCACCGCAGATAT |  |  |
| Cgsl-24F | TCGTACCACTGTGCTTATC | 667 | 52 |
| Cgsl-24R | TCGGCGTATTCTTCTTCAA |  |  |
| Cgsl-25F | GTTGTTAGGTTACCATCAATGTCC | 694 | 58 |
| Cgsl-25R | GCCATGTAGACTATATCAATCTCCT |  |  |
| Cgsl-26-28F | GCCGTAATCTACTTCCTTGT | 226 | 52 |
| Cgsl-26-28R | GAGAACACCGACATACAGAA |  |  |
| Cgsl-29F | TTCCAAGATGATGGCACCACATG | 293 | 60 |
| Cgsl-29R | TTACCATAGCTTGCTCCTGATCCTT |  |  |
| Cgsl-30F | ACTATCGTTACCTCGTGCTCTACAA | 500 | 60 |
| Cgsl-30R | ATCCATGCAGTTCCATTAGAGGGTA |  |  |
| Cgsl-31F | TCTTGGCGTGTTTATGTAGTGGGA | 371 | 60 |
| Cgsl-31R | ATCAGTGAAGATGGGCATAGGAACT |  |  |
| Cgsl-32F | GACTACAAACGCCTTTCAG | 118 | 52 |
| Cgsl-32R | GCAGCCAAGACATATCAC |  |  |
| Cgsl-33F | CTGTGTTGGATGTAGTGCCGTCGG | 387 | 62 |
| Cgsl-33R | GGTAGTGACCACCTCAAAGGACTCG |  |  |
| Cgsl-34F | TCAATGTGTCAGATAATGTTACT | 186 | 52 |
| Cgsl-34R | GACATAGAATGCTTAGGGTTT |  |  |
| Cgsl-35F | TGCTGTTATTGCTCGTTGCT | 102 | 58 |
| Cgsl-35R | TCGGCGTTGAGAGGACAAGT |  |  |
| Cgsl-36F | TTGTGTTACAGAGGATTGG | 188 | 52 |
| Cgsl-36R | CCTTGAGAATATCTTCACCTT |  |  |
| Cgsl-37F | TCGTACCTTCAGTGCTGGAGTATG | 445 | 60 |
| Cgsl-37R | ACAGTACCCAACATTCCCAAATCAG |  |  |
| Cgsl-38F | CTCGTTGCTCGGAATGAGATCGT | 381 | 60 |
| Cgsl-38R | TGCCGTGTGCAGACTGGTATG |  |  |
| Cgsl-39F | GTCCGGTTGGCCTAAAGTTG | 156 | 56 |
| Cgsl-39R | CCTTTTGTAATGACTTCCAGGGG |  |  |
| Cgsl-40F | AGTGGCACCAACATATCTAT | 159 | 52 |
| Cgsl-40R | AGCAGTATTCCAACCAGAA |  |  |
| Cgsl-41F | GACGACATCGACCCATTCTC | 127 | 56 |
| Cgsl-41R | TTAAGCGCCAACTGCCTATT |  |  |
| Cgsl-42-43F | TTGCGCCTTTTTCCTAGTTA | 369 | 54 |
| Cgsl-42-43R | CGCACCTTAAACTGAAGCAC |  |  |
| Cgsl-44F | CCCTCCTACAACCACTCCCAGAA | 562 | 60 |
| Cgsl-44R | TTCGTTCGCTGAATCATCCATCATT |  |  |
| Cgsl-45F | ACCCTTTAAGGTAACTCCATACTCG | 716 | 60 |
| Cgsl-45R | AATGAGCGAACGACAGAATCTTGAA |  |  |
| Cgsl-46-48F | TTATCAGTCGGTCGGAAGA | 355 | 58 |
| Cgsl-46-48R | CAGAGGACAACAGAAGCATA |  |  |
| Cgsl-49F | GCATGTTGCTTTGTATGAGGGCT | 537 | 60 |
| Cgsl-49R | CATTCGCAGGGGTGATAAACGC |  |  |
| Cgsl-50F | AGGGCACTTCTCTATCAGCACAAAC | 528 | 60 |
| Cgsl-50R | ACAGCGTAATTATCGTCCTCGTTCA |  |  |

**Table S**8 The information of loci selected for validation.

| **Loci** | **Genome locus** | **Start** | **End** | **Reference** | **Mutant** | **Variation type** | | **MM** | **FF** | **MF** | **FM** |
| --- | --- | --- | --- | --- | --- | --- | --- | --- | --- | --- | --- |
| Cgsl-1 | NW_011934793.1 | 82293 | 82293 | A | G | SNP | 0|1 | | 0|0 | 0|1 | 0|0 |
| Cgsl-2 | NW_011934804.1 | 85697 | 85697 | A | C | SNP | 0|1 | | 0|0 | 0|0 | 0|0 |
| Cgsl-3 | NW_011936003.1 | 246837 | 246837 | A | G | SNP | 0|1 | | 0|0 | 0|0 | 0|0 |
| Cgsl-4 | NW_011936396.1 | 38189 | 38189 | G | A | SNP | 0|1 | | 0|0 | 0|0 | 0|0 |
| Cgsl-5 | NW_011935314.1 | 55680 | 55680 | A | G | SNP | 0|1 | | 0|0 | 0|0 | 0|0 |
| Cgsl-6 | NW_011937092.1 | 54568 | 54568 | C | G | SNP | 0|1 | | 1|1 | 1|1 | 1|1 |
| Cgsl-7 | NW_011938059.1 | 439067 | 439067 | T | C | SNP | 0|1 | | 1|1 | 1|1 | 1|1 |
| Cgsl-8 | NW_011936984.1 | 850284 | 850284 | G | T | SNP | 0|1 | | 1|1 | 1|1 | 1|1 |
| Cgsl-9 | NW_011937325.1 | 52843 | 52843 | A | T | SNP | 0|0 | | 0|1 | 0|0 | 0|0 |
| Cgsl-10 | NW_011935359.1 | 133881 | 133881 | T | A | SNP | 0|0 | | 0|1 | 0|0 | 0|0 |
| Cgsl-11 | NW_011936434.1 | 170442 | 170442 | G | A | SNP | 0|0 | | 0|1 | 0|0 | 0|0 |
| Cgsl-12 | NW_011937556.1 | 52454 | 52454 | A | T | SNP | 0|0 | | 0|1 | 0|0 | 0|0 |
| Cgsl-13 | NW_011937548.1 | 291822 | 291822 | G | A | SNP | 0|0 | | 0|1 | 0|0 | 0|0 |
| Cgsl-14 | NW_011936459.1 | 59420 | 59420 | A | T | SNP | 1|1 | | 0|1 | 1|1 | 1|1 |
| Cgsl-15 | NW_011935873.1 | 48873 | 48873 | T | C | SNP | 1|1 | | 0|1 | 1|1 | 1|1 |
| Cgsl-16 | NW_011935873.1 | 49256 | 49256 | A | G | SNP | 1|1 | | 0|1 | 1|1 | 1|1 |
| Cgsl-17 | NW_011937770.1 | 504919 | 504919 | T | A | SNP | 0|0 | | 0|1 | 0|1 | 0|1 |
| Cgsl-18 | NW_011934623.1 | 8933 | 8933 | G | A | SNP | 1|1 | | 0|1 | 0|1 | 0|1 |
| Cgsl-19 | NW_011934983.1 | 254487 | 254487 | T | G | SNP | 1|1 | | 0|1 | 0|1 | 0|1 |
| Cgsl-20 | NW_011935062.1 | 20925 | 20925 | T | G | SNP | 0|0 | | .|. | 0|0 | 0|0 |
| Cgsl-21 | NW_011936103.1 | 146690 | 146690 | C | T | SNP | 1|1 | | 1|1 | .|. | .|. |
| Cgsl-22 | NW_011936103.1 | 146702 | 146704 | TAA | - | Deletion | 1|1 | | 1|1 | .|. | .|. |
| Cgsl-23 | NW_011936166.1 | 57933 | 57933 | A | T | SNP | 1|1 | | 1|1 | .|. | .|. |
| Cgsl-24 | NW_011937876.1 | 17819 | 17819 | C | T | SNP | 1|1 | | 1|1 | .|. | .|. |
| Cgsl-25 | NW_011937914.1 | 665831 | 665831 | T | C | SNP | 1|1 | | 1|1 | .|. | .|. |
| Cgsl-26 | NW_011936772.1 | 13182 | 13182 | C | T | SNP | 0|0 | | .|. | .|. | .|. |
| Cgsl-27 | NW_011936772.1 | 13206 | 13206 | C | A | SNP | 0|0 | | .|. | .|. | .|. |
| Cgsl-28 | NW_011936772.1 | 13214 | 13214 | G | T | SNP | 0|0 | | .|. | .|. | .|. |
| Cgsl-29 | NW_011934783.1 | 118672 | 118672 | - | GGGATCTTGGGGTTGC | Insertion | 1|1 | | .|. | .|. | .|. |
| Cgsl-30 | NW_011935279.1 | 627143 | 627148 | TATTAA | - | Deletion | 1|1 | | .|. | .|. | .|. |
| Cgsl-31 | NW_011935720.1 | 50233 | 50239 | TACAACG | - | Deletion | 1|1 | | .|. | .|. | .|. |
| Cgsl-32 | NW_011934994.1 | 399626 | 399626 | A | G | SNP | 0|1 | | 0|0 | 0|0 | 0|0 |
| Cgsl-33 | NW_011935585.1 | 124803 | 124803 | A | G | SNP | 0|1 | | 0|0 | 0|0 | 0|0 |
| Cgsl-34 | NW_011937453.1 | 56883 | 56883 | A | T | SNP | 0|0 | | 0|0 | 0|1 | 0|0 |
| Cgsl-35 | NW_011937968.1 | 36710 | 36710 | T | A | SNP | 0|0 | | 0|0 | 0|1 | 0|0 |
| Cgsl-36 | NW_011934885.1 | 366177 | 366177 | C | T | SNP | 0|0 | | 0|1 | 0|0 | 0|0 |
| Cgsl-37 | NW_011936632.1 | 144962 | 144962 | C | T | SNP | 0|0 | | 0|1 | 0|0 | 0|0 |
| Cgsl-38 | NW_011937582.1 | 119203 | 119203 | C | T | SNP | 0|0 | | 0|1 | 0|0 | 0|0 |
| Cgsl-39 | NW_011935992.1 | 1533314 | 1533314 | T | C | SNP | 1|1 | | 1|1 | 1|1 | 0|1 |
| Cgsl-40 | NW_011936629.1 | 576359 | 576359 | T | G | SNP | 0|1 | | 0|1 | 0|1 | 0|0 |
| Cgsl-41 | NW_011937064.1 | 660408 | 660408 | C | T | SNP | 0|1 | | 0|1 | 0|1 | 1|1 |
| Cgsl-42 | NW_011937600.1 | 70645 | 70645 | C | T | SNP | 0|1 | | 0|1 | 0|0 | 0|1 |
| Cgsl-43 | NW_011937600.1 | 70706 | 70706 | A | G | SNP | 0|1 | | 0|1 | 0|0 | 0|1 |
| Cgsl-44 | NW_011936540.1 | 20746 | 20746 | T | A | SNP | 1|1 | | .|. | 1|1 | 1|1 |
| Cgsl-45 | NW_011937022.1 | 31768 | 31768 | C | T | SNP | 1|1 | | .|. | 1|1 | 1|1 |
| Cgsl-46 | NW_011936103.1 | 714072 | 714072 | A | G | SNP | 0|0 | | 1|1 | .|. | .|. |
| Cgsl-47 | NW_011936103.1 | 714086 | 714086 | A | T | SNP | 0|0 | | 1|1 | .|. | .|. |
| Cgsl-48 | NW_011936103.1 | 714104 | 714104 | T | C | SNP | 0|0 | | 1|1 | .|. | .|. |
| Cgsl-49 | NW_011937466.1 | 18993 | 18995 | AAT | - | Deletion | .|. | | 1|1 | .|. | .|. |
| Cgsl-50 | NW_011937624.1 | 23651 | 23651 | - | GGA | Insertion | .|. | | 1|1 | .|. | .|. |

0|0 and 1|1: homozygote; 0|1: heterozygote; .|.; deletant. The loci marked in red are these where sanger sequencing results kept consistent with RAD-seq. The underlined SNPs were confirmed correct by Sanger sequencing.


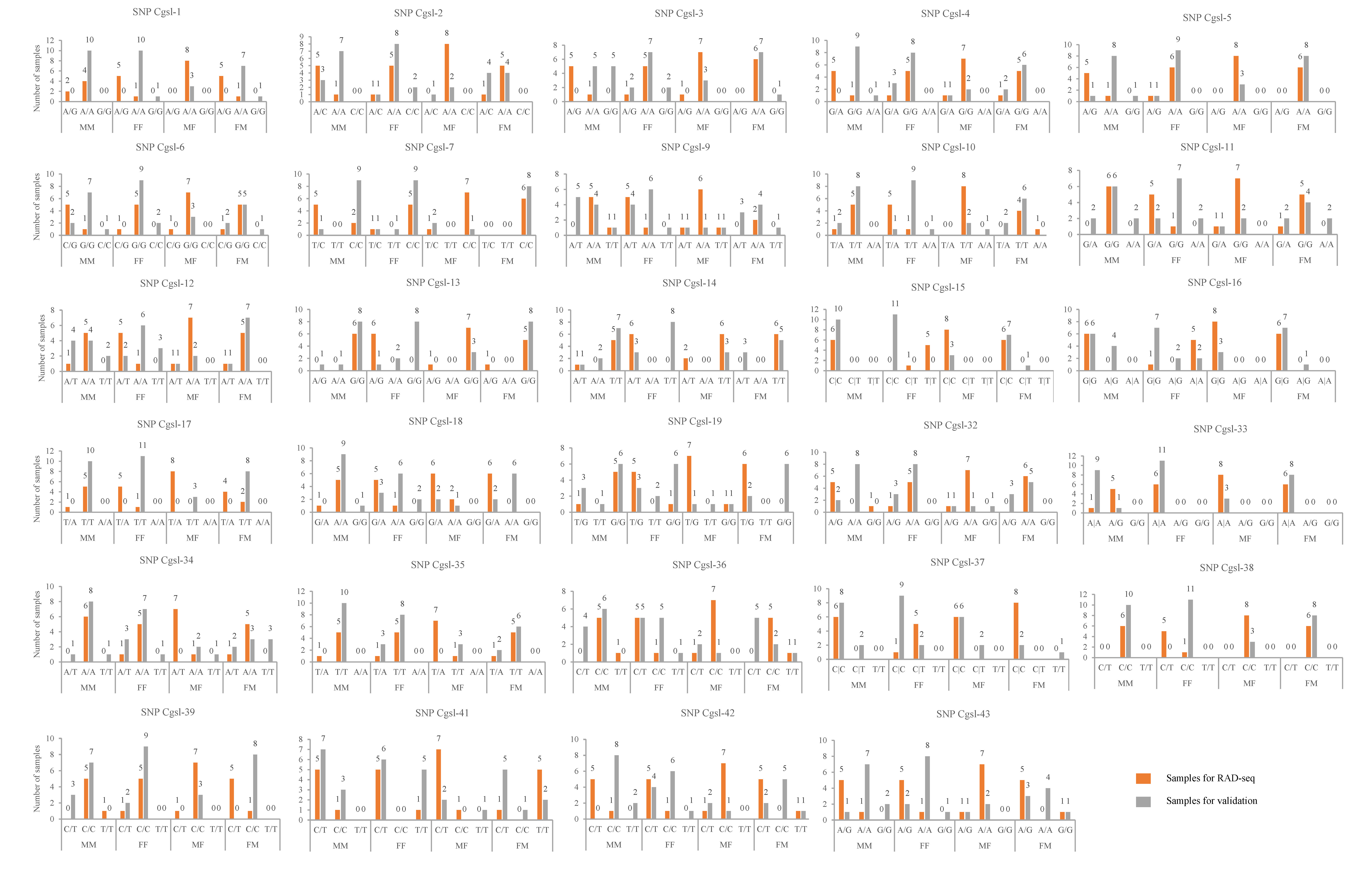


Figure S1 Numbers of individuals with different genotypes at 29 SNPs. The horizontal axis represents genotype of individuals in the four phenotype groups. The orange and grey column represent samples for RAD-seq and validation, respectively. And the numbers of oysters are reported upon the column.
